# Retrieval of retrained and reconsolidated memories are associated with a distinct neural network

**DOI:** 10.1038/s41598-018-37089-2

**Published:** 2019-01-28

**Authors:** Luz Bavassi, Cecilia Forcato, Rodrigo S. Fernández, Gabriela De Pino, María E. Pedreira, Mirta F. Villarreal

**Affiliations:** 1 Universidad de Buenos Aires, Facultad de Ciencias Exactas y Naturales, Departamento de Física, Ciudad de Buenos Aires, Argentina; 2 CONICET-Universidad de Buenos Aires, Instituto de Fisiología, Biología Molecular y Neurociencias (IFIBYNE), Ciudad de Buenos Aires, Argentina; 3 Unidad Ejecutora de Estudios de Neurociencias y Sistemas Complejos, CONICET, Universidad Nacional Arturo Jauretche Hospital de Alta Complejidad en Red El Cruce “Néstor Kirchner”,Av. Calchaqui 6200, (1888) Florencio Varela, Argentina; 4 Universidad de Buenos Aires, Facultad de Ciencias Exactas y Naturales, Ciudad de Buenos Aires, Argentina; 5 Laboratorio de Neuroimágenes, Departamento de Imágenes, FLENI, Montañeses 2325, Ciudad de Buenos Aires, C1428AQK Argentina; 6 Centro Universitario de Imágenes Médicas (CEUNIM), Escuela de Ciencia y Tecnología, Universidad Nacional de San Martín, Buenos Aires, Argentina; 7 INAAC, FLENI, Montañeses 2325, C1428AQK Ciudad de Buenos Aires, Argentina; 8 CONICET, Ciudad de Buenos Aires, Argentina; 9 Present Address: Instituto de Fisiología, Biología Molecular y Neurociencias, Facultad de Ciencias Exactas y Naturales, Universidad de Buenos Aires, Ciudad Universitaria (C1428EHA), Ciudad de Buenos Aires, Argentina

## Abstract

Consolidated memories can persist from a single day to years, and persistence is improved by retraining or retrieval-mediated plasticity. One retrieval-based way to strengthen memory is the reconsolidation process. Strengthening occurs simply by the presentation of specific cues associated with the original learning. This enhancement function has a fundamental role in the maintenance of memory relevance in animals everyday life. In the present study, we made a step forward in the identification of brain correlates imprinted by the reconsolidation process studying the long-term neural consequences when the strengthened memory is stable again. To reach such a goal, we compared the retention of paired-associate memories that went through retraining process or were labilizated-reconsolidated. Using functional magnetic resonance imaging (fMRI), we studied the specific areas activated during retrieval and analyzed the functional connectivity of the whole brain associated with the event-related design. We used Graph Theory tools to analyze the global features of the network. We show that reconsolidated memories imprint a more locally efficient network that is better at exchanging information, compared with memories that were retrained or untreated. For the first time, we report a method to elucidate the neural footprints associated with a relevant function of memory reconsolidation.

## Introduction

Consolidated memories can persist from 24 hours to years, depending on the saliency, intensity or emotionality of the information to be remembered^1^. It is an accepted idea that the mechanisms responsible for making a memory long-lasting must persist to make the trace resistant to forgetting^2–5^. It is possible to modify the persistence of a memory after consolidation simply by retraining or by the presentation of a reminder. A reminder is defined as a group of cues that evoke the original memory trace^6,7^. An example of a reminder based way to strengthen memory is the reconsolidation process. That is, when consolidated memories are reactivated by the presentation of specific reminders, the retrieved memory enters in a period of labilization followed by a process of restabilization known as reconsolidation^8–10^. Memory reconsolidation has a fundamental role in the maintenance of memory relevance^11^. This process is in charge of modifying the strength and/or content of consolidated memories. It has been observed in animal models and in human experiments^12–15^. Depending on the treatment used in the reactivation session, memories can be strengthened, weakened, or updated^16^. Retention can be enhanced by the administration of various compounds after reactivation or after recurrent presentations of a specific reminder^17^. Importantly, not every reminder is capable of inducing memory labilization-reconsolidation. Thus, reminders with an incongruence between actual and past events, a form of “prediction error”, are most effective triggering memory destabilization of the target trace^18,19^. The destabilization-reconsolidation process has been reported after the presentation of salient training clues as the context or the unconditioned stimulus in a pavlovian association task^18^ or the presentation of a fraction of a movie in a episodic memory protocol^19^. Particularly, in a declarative memory task named syllable pair protocol we demonstrated that reconsolidation only occurs with the presentation of an ‘incomplete reminder’, i.e., a reminder that did not include all cues of the original learning sequence^13^. We showed that learning a new task after the presentation of the incomplete reminder affected memory restabilization, but learning the new task not affected memory after the presentation of a complete reminder^20^. In order to elucidate reconsolidation functions, we showed that the presentation of more than one incomplete reminder improves the performance in the testing session by enhancing memory precision^21^. We also demonstrated that only one labilization-reconsolidation process triggered by an incomplete reminder strengthens the original memory, increasing its persistence and making it more resistant to forgetting. The effect, of only one incomplete reminder, was observed seven days after training but not three days after learning, probably due to a ceiling effect^21^.

The reconsolidation process has been well-studied at a behavioral and cellular level. Recently, studies have advanced in the understanding of the neural mechanisms involved in the reconsolidation process in humans using neuroimaging techniques mainly focusing on the brain correlates of memory reactivation^22–25^. In a previous report we made a step forward in understanding the neural markers of the reconsolidation process by adapting the syllable pair protocol to be used as an intra-subject fMRI task named picture-word association task^26^. As in the syllable pair protocol, this task consisted of different types of reminders: the cue-reminder, an incomplete reminder that included a picture plus the first syllable of its associated word (Rc); and the word reminder, a complete reminder that included a picture plus the full-word associated (Rw). As in the syllable pair protocol, we enlighten the reconsolidation process because memory- restabilization was impaired with a new learning task. Moreover, we observed that the presentation of the incomplete reminder (Rc) improved the performance of individuals at testing session five days after training. This enhancement also appeared for the retrained words, the ones that received the complete reminder during the treatment session. These results support the idea that both, the reactivation followed by restabilization of the consolidated memory and a retraining treatment, strengthen the retention of the original memory trace.

Currently, it is still not clear which changes induced by memory reactivation could persist in the long term^27^. However, if a mnemonic trace can be strengthened by reminder-based processes, this may imply neural rearrangement in brain areas related to this memory, and these changes might persist after the reactivation session. In other words, we expect that the enhancement of a memory by reconsolidation will leave neural footprints. Furthermore, we expect differences between neural markers imprinted by the labization-restabilization process and retraining. In this report, using the picture-word association task^26^, we were interested in exploring the neural consequences of memory strengthening in a long term test. The task included three sessions: during the first session (training) participants have to learn 36 pairs of pictures and words; at the second session (reactivation, 48 hours later) they received different types of reminders that were intended either to retrain the memory trace (Rw, complete reminder, similar to the training session), to reactivate and reconsolidate it (Rc, the incomplete reminder), or not to reactivate it (Nr); finally, the memory retention was evaluated during the third session (testing). In a behavioral experiment, we compared the efficacy of these reminders in the maintenance of the memory trace in an early and a delayed test; in a second step, in an fMRI experiment, we analyzed the neural circuits involved in the retrieval of memories that were previously reconsolidated, retrained or not recalled in both time points.

Considering that we expected subtle differences between conditions in isolated brain regions activity during the retrieval of a associative declarative memory, we proposed a novel analysis studying the interactions of the whole brain during the retrieval. Different higher cognitive tasks share changes of activity in individual regions, in consequence the final behavior might be due to the activity and the interactivity of the network. Moreover, it has been proposed that the diversity of memory processes and phenomenons are features that emerge from the interaction among brain regions^28^. In the present study, we used Graph Theory tools to analyze the functional connectivity of the task-related network^29–31^. This mathematical tool provides a common language for the analysis of complex systems, and it is used to describe some of the key topological properties^32^, being useful to compare special features of different networks such as the high dimensional interconnected topology. Taking into account previous neuroimaging results of neural signatures related with retrieval of episodic memory which reported (a) an increased of activity of the amygdala and hippocampus associated with reduced of the subsequent memory in a emotional picture reconsolidation protocol^33^ and (b) a less segregated modular neural network related with the successfully remembered words in a short-term task^34^. We expect to find differences in the architecture of the networks generated for reconsolidated, retrained and no remembered items. We hypothesize that the reconsolidated pairs of pictures and words are more resistant to forgetting and that the retrieval of these memories are associated with a brain network more connected and better in the exchange of information.

## Results

### Behavioral experiment

**Figure 1:**
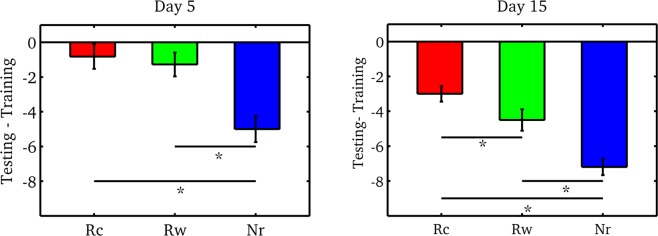
Behavioral experiment - Memory retention on day 5 and on day 15. We studied the memory retention five days and fifteen days after training (left and right panel, respectively). We defined retention as the difference between correct responses at testing and training for the different conditions of reminders (Rc: red, Rw: green and Nr: blue). (MEAN ± SEM) *P < 0.05.

The aim of this intra-subject design was to characterize the post-treatment effect of a memory that passed through different processes: reconsolidation (Rc, incomplete reminder), retraining (complete reminder, similar to the training session, Rw) or no treatment (Nr). We compared the retention (difference between the number of correct responses at testing and the number of correct responses at training) in a group five days after training and in a different group fifteen days after memory acquisition, Fig. 1. In this context, we performed a repeated-measures ANOVA with “reminder condition” (Rc, Rw, Nr) as within-subjects factor and “time” (Day 5 and Day 15) as the between-subjects factor. This analysis showed a main effect of reminder condition (Group factor: F(2,44) = 47.8, P < 0.001, ηp^2^ = 0,64) and a main effect for evaluation time (F(1,22) = 17.5, P < 0.001, ηp^2^ = 0,32) but not significant interaction (F(2,44)  = 1.5, P > 0.05). As expected, in the testing session on day 5 (left panel, Fig. 1) Rc and Rw showed a better retention than Nr. The post-hoc pairwise comparisons between Rc and Nr and between Rw and Nr were significant (P_all_ < 0.001) while it was not possible to distinguish between the Rc and Rw (P > 0,05). On the other hand, we could differentiate between the three conditions when the testing session was further away in time from training (right panel, Fig. 1, every post-hoc pairwise comparison was significant Ps < 0.05). On day 15, associations that went through the reconsolidation process (Rc) had the best performance while the Nr condition presented the lowest retention.

The learning level did not influence performance in the evaluation sessions. There were no significant differences between conditions in the number of correct responses. The mean correct responses were Rc = 9.5 ± 0.5, Rw = 9.3 ± 0.5 and Nr = 9.3 ± 0.6 for the group that was evaluated five days after training and Rc = 9.3 ± 0.7, Rw = 9.5 ± 0.5 and Nr = 9.2 ± 0.5 for day 15 group.

As we expected, both Rc and Rw treatments during the reactivation session enhanced memory, making it last longer than memories that were not reactivated. In addition, these results showed that it was not possible to differentiate between these conditions in the early test (day 5), but when the testing was far away in time from the training (day 15), memories that passed through the reconsolidation process (Rc) were more resistant to forgetting compared to Rw and Nr, which led to a better performance.

### fMRI experiment

**Figure 2:**
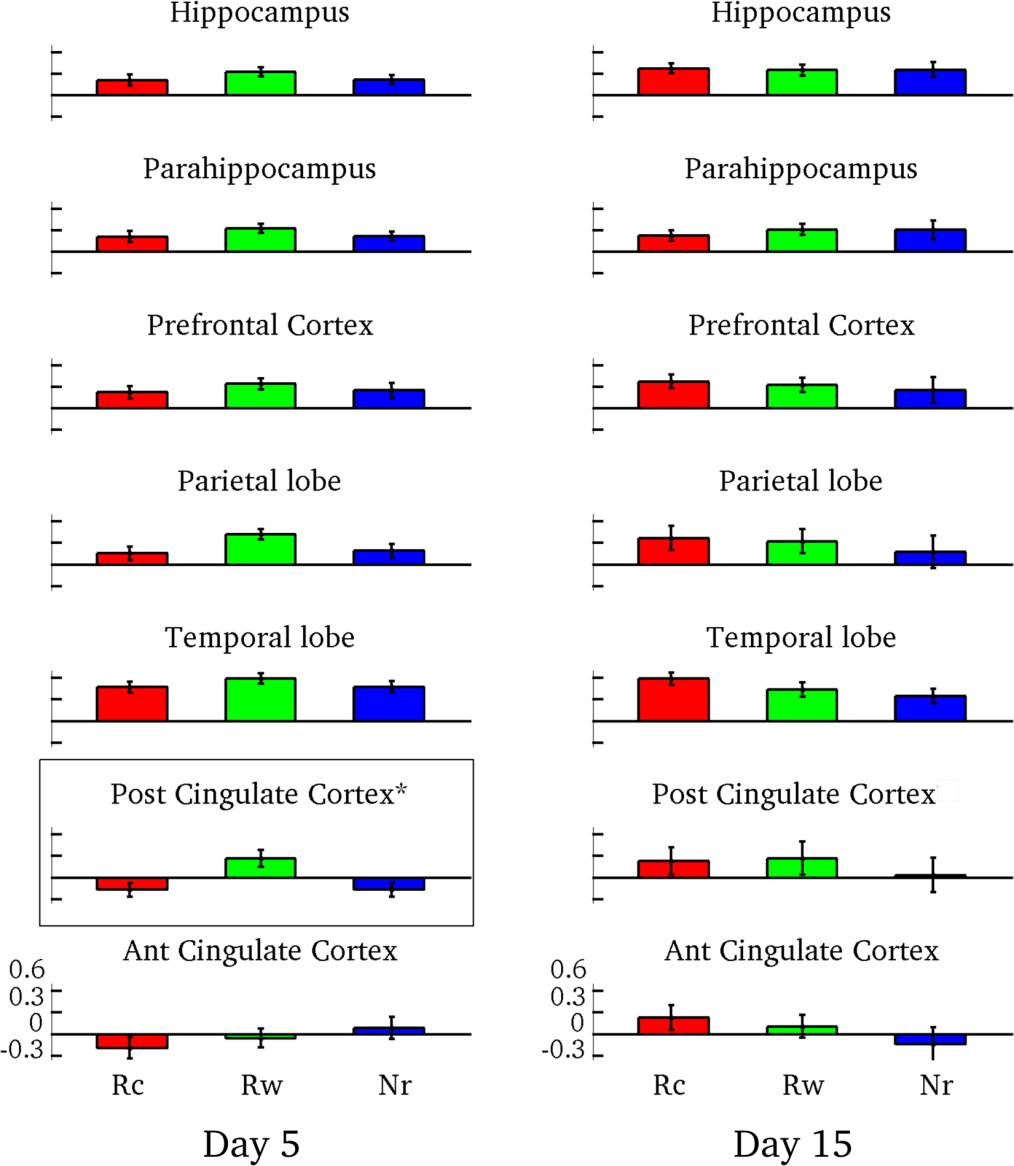
fMRI experiment - ROIs analysis on day 5 and on day 15. β-values for seven bilateral big regions: hippocampus, parahippocampus, prefrontal cortex, parietal lobe, temporal lobe, posterior cingulate cortex and anterior cingulate cortex for day 5 and day 15 testing session. Only the posterior cingulate cortex presented significant difference on day 5, *P < 0.05.

As shown in the previous experiment, a memory that passed through the reconsolidation process is maintained longer than another that was retrained. In order to find the neural consequences of memory strengthening we repeated the same experiment, performing the testing session inside an fMRI scanner. After the testing session, outside the scanner, participants filled a written form reporting the words said inside the scanner. The questionnaire results were similar to the ones obtained in the behavioral experiment. In this experiment the learning rate was not significantly different between conditions: Rc = 9.8 ± 0.4, Rw = 9.3 ± 0.6 and Nr = 9.2 ± 0.4 for the five days group and Rc = 9.1 ± 0.6, Rw = 9.4 ± 0.5 and Nr = 9.4 ± 0.4 for the delay group. To explore the neural imprint of a memory that was strengthened, we compared the BOLD signal (blood oxygen level dependent) between conditions during the testing session at day 5 and at day 15. First, we analyzed the beta values of 32 ROIs between conditions, Table 1. These regions were selected because they are known to be involved in the successful retrieval of episodic memories^35,36^ and/or were found in a whole brain analysis comparing pairs of conditions (Supplementary Fig. S1 and Table S2). Left and right posterior cingulum showed significant differences on day 5, Rw was the highest beta value, Table 1. Although there were no other significant differences on day 5, the Rw presented a trend toward greater activity than the other two conditions. For sake of simplicity, we pooled the 32 ROIs in 7 big bilateral regions: hippocampus, parahippocampus, prefrontal cortex, parietal lobe, temporal lobe, posterior cingulum and anterior cingulum, Fig. 2. The Rw value on day 5 was the highest in six big regions although the difference was only significant in the posterior cingulum region (F(2,39) = 5.67, P < 0.01, left column Fig. 2). On the other hand, on day 15, none of the regions showed significant differences between conditions (Table 1). Moreover, Rw and Rc conditions seem to have similar activity levels in the seven big regions (P > 0.05, right column Fig. 2).

**Table 1:** Rois beta values.

	Day 5				Day 15			
	Rc	Rw	Nr	p	Rc	Rw	Nr	p
Left hippocampus	0.20 ± 0.08	0.30 ± 0.06	0.23 ± 0.07	0.61	0.39 ± 0.08	0.36 ± 0.08	0.37 ± 0.12	0.98
Right hippocampus	0.23 ± 0.05	0.35 ± 0.05	0.20 ± 0.05	0.25	0.35 ± 0.07	0.33 ± 0.08	0.34 ± 0.01	0.98
Left parahippocampal	0.19 ± 0.07	0.27 ± 0.04	0.17 ± 0.06	0.60	0.22 ± 0.06	0.32 ± 0.08	0.31 ± 0.14	0.73
Right parahippocampal	0.24 ± 0.05	0.34 ± 0.05	0.17 ± 0.06	0.20	0.23 ± 0.10	0.30 ± 0.09	0.30 ± 0.12	0.87
Left frontal inferior triangularis	0.49 ± 0.12	0.62 ± 0.07	0.47 ± 0.13	0.63	0.60 ± 0.13	0.48 ± 0.11	0.56 ± 0.14	0.80
Right frontal inferior triangularis	0.41 ± 0.09	0.58 ± 0.07	0.51 ± 0.12	0.54	0.56 ± 0.17	0.43 ± 0.14	0.52 ± 0.11	0.81
Left frontal superior medial	0.07 ± 0.13	0.18 ± 0.13	0.12 ± 0.14	0.84	0.30 ± 0.15	0.26 ± 0.16	0.28 ± 0.29	0.99
Right frontal superior medial	−0.02 ± 0.11	0.11 ± 0.10	0.09 ± 0.15	0.71	0.30 ± 0.15	0.28 ± 0.14	0.24 ± 0.28	0.98
Left frontal superior	0.16 ± 0.10	0.25 ± 0.08	0.18 ± 0.09	0.78	0.27 ± 0.10	0.21 ± 0.10	0.09 ± 0.21	0.69
Right frontal superior	0.14 ± 0.08	0.21 ± 0.08	0.13 ± 0.12	0.82	0.27 ± 0.09	0.21 ± 0.08	0.09 ± 0.19	0.61
Left frontal middle	0.32 ± 0.11	0.45 ± 0.10	0.32 ± 0.12	0.60	0.43 ± 0.12	0.39 ± 0.11	0.23 ± 0.25	0.70
Right frontal middle	0.34 ± 0.09	0.46 ± 0.10	0.35 ± 0.13	0.68	0.42 ± 0.12	0.34 ± 0.12	0.19 ± 0.21	0.58
Left middle orbital frontal	0.13 ± 0.11	0.24 ± 0.09	0.13 ± 0.10	0.69	0.30 ± 0.09	0.30 ± 0.09	0.21 ± 0.11	0.74
Right middle orbital frontal	0.20 ± 0.13	0.30 ± 0.11	0.18 ± 0.08	0.71	0.28 ± 0.13	0.32 ± 0.11	0.09 ± 0.13	0.40
Left supramarginal	0.43 ± 0.09	0.50 ± 0.08	0.38 ± 0.12	0.72	0.48 ± 0.14	0.30 ± 0.20	0.29 ± 0.19	0.70
Right supramarginal	0.21 ± 0.06	0.28 ± 0.06	0.17 ± 0.10	0.66	0.28 ± 0.14	0.32 ± 0.15	0.11 ± 0.14	0.55
Left angular	-0.28 ± 0.16	0.17 ± 0.11	-0.15 ± 0.12	0.08	−0.02 ± 0.23	0.04 ± 0.18	−0.25 ± 0.35	0.73
Right angular	−0.22 ± 0.13	0.18 ± 0.12	−0.09 ± 0.13	0.07	0.17 ± 0.20	0.24 ± 0.15	0.01 ± 0.23	0.71
Left parietal superior	0.51 ± 0.14	0.81 ± 0.12	0.48 ± 0.12	0.16	0.68 ± 0.27	0.54 ± 0.26	0.46 ± 0.32	0.87
Right parietal superior	0.28 ± 0.13	0.57 ± 0.13	0.37 ± 0.12	0.29	0.62 ± 0.28	0.50 ± 0.28	0.41 ± 0.30	0.88
Left temporal superior	0.96 ± 0.11	1.04 ± 0.12	0.98 ± 0.13	0.90	0.98 ± 0.15	0.75 ± 0.14	0.59 ± 0.16	0.21
Right temporal superior	0.71 ± 0.10	0.84 ± 0.08	0.73 ± 0.10	0.60	0.78 ± 0.11	0.73 ± 0.13	0.55 ± 0.16	0.47
Left temporal middle	0.40 ± 0.09	0.51 ± 0.09	0.39 ± 0.10	0.66	0.55 ± 0.11	0.37 ± 0.12	0.26 ± 0.12	0.28
Rigth temporal middle	0.31 ± 0.11	0.48 ± 0.07	0.34 ± 0.08	0.36	0.45 ± 0.08	0.32 ± 0.12	0.29 ± 0.11	0.54
Left inferior temporal	0.19 ± 0.06	0.32 ± 0.06	0.18 ± 0.05	0.19	0.39 ± 0.08	0.23 ± 0.09	0.13 ± 0.10	0.14
Right inferior temporal	0.24 ± 0.08	0.36 ± 0.07	0.22 ± 0.07	0.40	0.35 ± 0.10	0.23 ± 0.08	0.25 ± 0.08	0.61
**Left posterior cingulum**	**−0.24 ± 0.09**	**0.21 ± 0.12**	**−0.26 ± 0.11**	**0.05**	0.19 ± 0.17	0.20 ± 0.22	−0.03 ± 0.25	0.68
**Right posterior cingulum**	**−0.10 ± 0.09**	**0.31 ± 0.12**	**−0.08 ± 0.09**	**0.01**	0.27 ± 0.21	0.34 ± 0.24	0.11 ± 0.23	0.76
Left anterior cingulum	−0.19 ± 0.14	−0.048 ± 0.13	0.07 ± 0.14	0.44	0.22 ± 0.18	0.11 ± 0.17	−0.21 ± 0.23	0.29
Right anterior cingulum	−0.18 ± 0.15	−0.06 ± 0.12	0.10 ± 0.15	0.41	0.24 ± 0.17	0.11 ± 0.14	−0.07 ± 0.23	0.50

**Figure 3:**
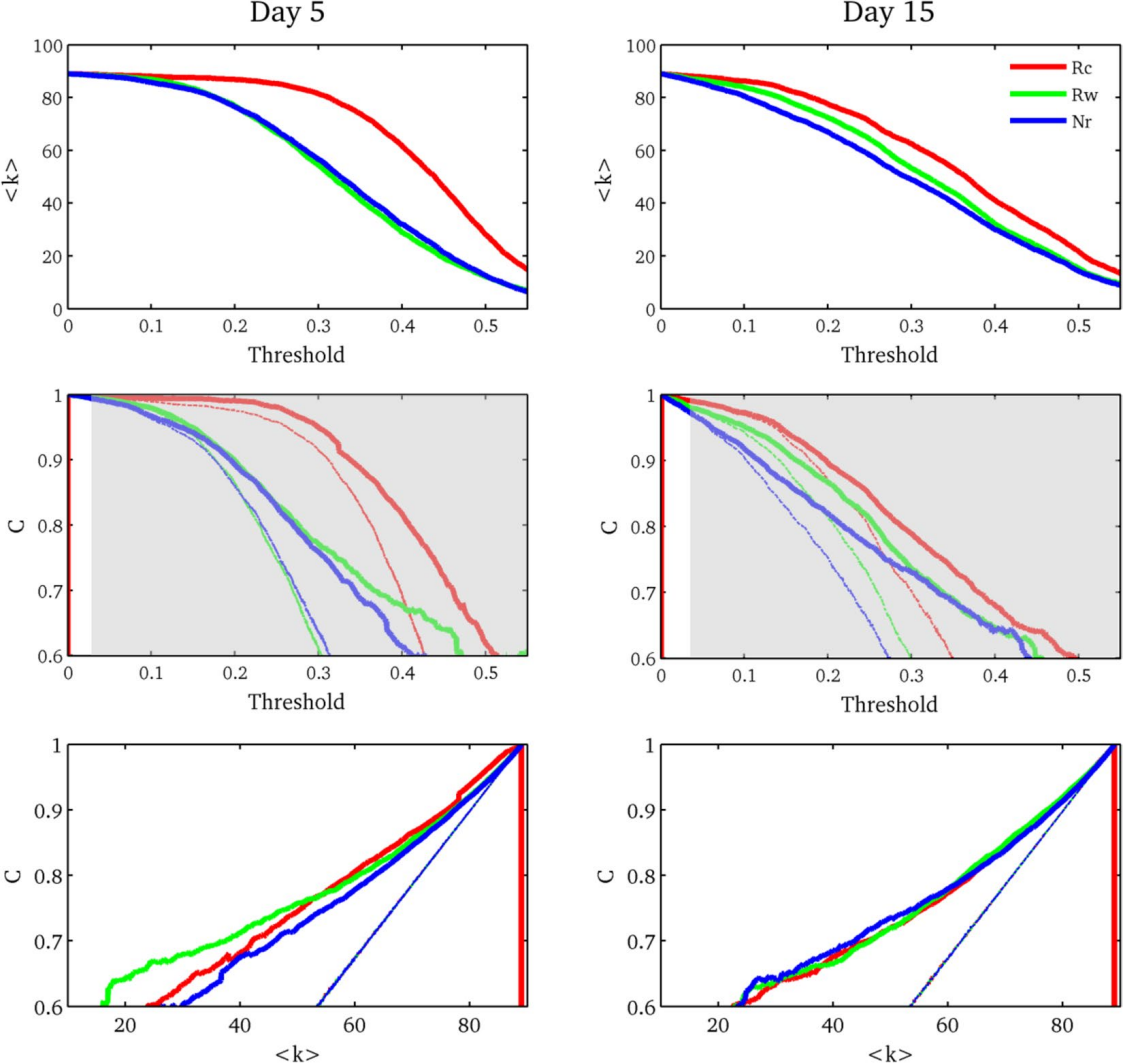
fMRI experiment - Graph Theory measures for both testing sessions. (Upper panel) Average degree (<k>) as a function of threshold for the three conditions. (Middle panel) Clustering size (C) as a function of threshold for the three conditions (thicker lines) and random graphs (thinner lines). The gray shadow represents threshold values where C for the conditions was significantly different of the random one. (Lower panel) Clustering size as a function of the average degree for the three conditions (thicker lines) and random graphs (thinner lines). Rc: red, Rw: green and Nr: blue.

To understand the neural organization after a treatment that enhanced memory, we explored the architectural features of the brain network per condition during the testing session at two time points. Thus, we performed the beta time series analysis for Rc, Rw and Nr conditions described in Materials and Methods. To have a “bird’s eye view” of the features of each network, we studied the average degree (<k>, average of number of links per node) and the clustering coefficient (C, number of triangles per node) for different threshold values, Fig. 3 (day 5: left column; day 15: right column). For both testing sessions, the three conditions showed the same pattern, but the difference between the curves in the delay test were smaller (Fig. 3). Rc average degree (<k>, red filled curve) as a function of threshold differed from Rw and Nr measurements (green and blue filled curves, respectively). Particularly, on day 5, the graph measures of the Rw were similar to the Nr networks, although small differences appeared on the delay test between these two conditions (upper and middle panels, Fig. 3). <k> decreased when the threshold increased (Fig. 3: upper panels). A lower threshold implies a more connected network, and a higher threshold means a small graph with a low number of links. Interestingly, the curve of <k>for the Rc condition was higher than the ones for Rw and Nr. The middle panels of Fig. 3 present the clustering coefficient for both test sessions. Again, the Rc curve differed from the other two conditions on both tests. In both cases, the clustering coefficient was higher for Rc network, meaning that these graphs were more locally connected (denser). We also calculated C for random graphs with the same number of nodes and links per condition per threshold value (dashed lines, middle panels, Fig. 3). The shadow area represents the threshold values for the three conditions where C was significantly different from the random ones (P < 0.01 for threshold > 0.02 for Day 5 and threshold > 0.03 for Day 15). Finally, we plotted C as a function of <k>to determine whether the differences in C between conditions were due to the architecture of the graph or because of the number of nodes and links (lower panels, Fig. 3). For both tests, the C curves of Rc, Rw and Nr looked similar (thicker curves), and the three of them were higher than the ones of the random graphs (thinner lines), meaning more connected nodes.

**Figure 4:**
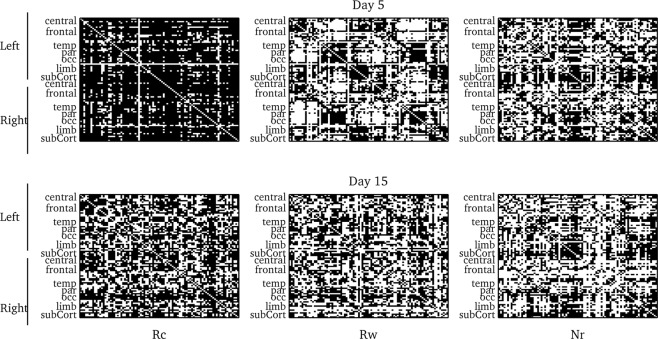
fMRI experiment – Adjacency matrices for 0.35 threshold. Adjacency matrix per condition for testing session 5 days after training (upper line) and for 15 days after training (lower line). ROIS were arranged by localization and lateralization.

We calculated the binary Adjacency matrices for a 0.35 threshold for the three conditions and both testing sessions, Fig. 4. The Rc matrix for day 5 testing session showed more black pairs meaning a denser graph (<k> = 74.8, C = 0.9), the links between ROIs were unspecific with no lateral predominance, Fig. 4 upper left panel. Beside, the Rw network (<k> = 42.3, C = 0.7) and Nr network (<k> = 44.9, C = 0.7) for the late test showed similar number of links and clustering coefficient, Fig. 4 upper middle and left panels. The number of links decreases in the delay test in the three conditions and the connections looked uniform distributed all over the brain, lower panels Fig. 4. The Adjacency matrix for Rc (<k> = 53.6, C = 0.8) still had a higher number of links while the clustering coefficient is quite similar to the ones of the Rw and Nr conditions (Rw: <k> = 44.6, C = 0.7 and Nr: <k> = 40.6,C = 0.7).

To sum up, we detected differences in the ROIs analyses on the testing session five days after training, with higher beta values in the posterior cingulum cortex for Rw (complete reminder) compared to Rc (incomplete reminder) and Nr conditions that were not present in the delay test. We also detected differences in the Rc network average degree (<k>) on both testing days. The Rc network was denser with unspecific links, with more nodes and links, showing a higher clustering coefficient per threshold. These differences were greater in the early test session.

## Discussion

Our main hypothesis was that there are differences in the imprinted brain changes in memory strengthening by reconsolidation or by retraining the original trace, and that those changes persist in the long term. For that, we performed a protocol were participants had to learn 36 pairs of pictures and words in the first session, 48 h later these associations were reactivated with three different treatments: an incomplete reminder that triggers the reconsolidation process (Rc), a complete reminder that was similar to the training session (Rw) and a no reminder condition (Nr); finally, five or fifteen days after training memory was evaluated. When memory was tested on day 5, we found that both the reconsolidated and retrained memory conditions (Rc and Rw, respectively) performed similarly among them but better than the untreated condition (Nr). However, the reconsolidated memories presented a denser network (larger average degree and clustering coefficient) than the retrained or untreated condition. These results might indicate that the reconsolidation process imprint a network that involves more regions more interconnected between them, based on the only difference between Rc and Rw conditions was the reminder treatment. Moreover, we found that the beta values of Rw were the highest of the three conditions in the posterior cingulum and that this trend was also observed in other ROIs. On the other hand, the testing session of an older memory (day 15) showed different results. As expected, reactivation strengthened memory in the Rc preventing it from being forgotten compared to the retrained items (Rw) which showed a poorer retention. Notably, these differences did not carry over to the images of neural activity during the retrieval of an older memory. When the testing session was further away in time from the training session, the ROI analysis showed no differences between conditions. Again, the Rc network was denser than the Rw and Nr ones, but the differences were smaller than the one obtained in the early testing.

The ROI results from day 5 showed that the Rw condition had higher beta values in six of the seven regions: hippocampus, parahippocampus, prefrontal cortex, parietal lobe, temporal lobe and posterior cingulum. Schwabe *et al*.^33^ reported higher BOLD activity in the hippocampus and amygdala associated with poorer memory retention in the evocation of emotional pictures. The authors suggested that the increased of the activity is related with the effort done in memory retrieval. In this sense, we might interpret that more effort is needed to evoke Rw pairs than Rc ones to achieve the same retention. The only region that was statistically different was the posterior cingulum. This region has been proposed to be the midline core of the default network^37^ and has a high baseline metabolic rate. Despite the central role it plays, there is no consensus about its functions, although its activity is known to be related to cognitive load in healthy brains^38^. In contrast, the scenario was not the same for the delay test. There were no differences between Rc and Rw conditions in the ROI analysis, perhaps due to the interaction with forgetting (poorer retention). The associations that were reconsolidated (Rc) were more resistant to the passage of time than the retrained ones (Rw). We can speculate that at the moment of the testing sessions, the information have already migrated to cortical areas, mostly independent of the hippocampus^39^.

Based on the hypothesis that behavioral differences between the conditions are a consequence of the interactions of the whole brain we explored the task-related network. We performed a deep description of the properties of the graphs. To obtain a broader picture of the architecture of the network and assuming that the threshold value has not a direct neurobiological interpretation, we explored the average degree and the clustering coefficient for many threshold values^40^. At day 5, for all threshold values, we found that the network associated with the retrieval of reconsolidated memories had the greatest average degree and clustering coefficient. However, these effects were more subtle at day 15 probably due to the passage of time. The networks of the three conditions and both evaluation session had a significant higher clustering coefficient than the random graph. This result implies that the networks involved in the retrieval process are more locally connected than a random graph with the same number of nodes,this higher interaction suggests a more fluent communication among regions and a better information transfer as a consequence of the treatment^32^. It is important to highlight that the clustering differences between conditions were a consequence of network size; the Rc network was larger than the other two. To sum up, we found that the brain network involved in the evocation of a memory that passed through the reconsolidation process is denser than the network of a memory that was retrained. If we consider that an efficient network is such that is good in exchanging information, then the Rc would be the most efficient one. It is now well established that memory traces are transformed over time. This modification may occur using different ways such as the strengthening of some traces by synaptic re-scaling during sleep^41,42^, the assimilation of new information into existing store information and the establishment of new linkages within existing memory traces^43^. In this framework we offer a possible explanation to present results. Reconsolidated items are supported by a wider network. This dimension would be explained by a memory transformation, in this case strengthening, which takes advantage of the new linkages and assimilation to previous knowledge stored in different regions. The assumption of the Rc network as being the more efficient one is in line with a recent work by Geib *et al*.^34^ that show, using another set of graph measures and only one threshold value, that the network involved in successful remembering of episodic memories was more integrative (higher global efficiency) compared with the network associated with forgetting. The graph features for the delay test were not that clear, probably due to the low number of correct responses.

Linking both results, the connectivity analysis and the ROI study, we proposed that the Rc network has more links distributed among the brain and this is counterbalanced with a lower activity in individual regions, suggesting that the energy saved in the BOLD activity is used in maintaining a denser graph.

Our results make a step forward in identifying neural footprints in memories enhanced by different reminder treatments: reconsolidation and retraining. The main function of reconsolidation is to update the content or the strength of the original trace. Though the importance of understanding how this process works, the strengthening function is scarcely explored in animals model^44,45^ and in humans^46–50^. Using an inhibitory avoidance task in rats, Inda and co-workers found that brief, non-reinforced context re-exposures strengthened memories through reconsolidation. Author reported that three context reactivations enhanced fear memories by engaging the direct functional connectivity between the dorsal hippocampus and the prelimbic cortex. Moreover, the authors found a critical involvement of BDNF and neuroligin 1 and 2 in promoting memory strengthening while inhibiting new learning (extinction)^44^. Regarding humans, Wichert and colleagues explored the possibility of altering retrieved episodic memories in relation to the age of memories^51^. Participants learned a set of emotional and neutral pictures and recalled them 1, 7, or 28 days later. The authors found that memory retrieval per se enhanced 28-day-old memories but had no effect on 1-day- or 7-day-old memories. Moreover, in motor memories, Wymbs and colleagues found that a skill could be strengthened by reconsolidation using a reactivation session followed by practice with a modified version of the skill with increased variability^49,52^.

We consider that the central framework for our results is the reconsolidation process, based on previous reports findings that demonstrated that the presentation of an incomplete reminder triggers the labilization-restabilization process and that its presence, without any manipulation, strengthens the target memory^26,48^. However, there might be alternative explanations for these results. Retrieval, the act of making stored information available for use, plays a central role in later recall. For example, it is known that taking a test improves later retention of the information compared with restudying the same material^53^. This testing effect could enrich existing memory traces or facilitate the access to the store information^54^. Commonly the treatment occurs near the acquisition, and the effect is evaluated at both short and long term test. In general, the testing effect considers exclusively the role of processing taking place during retrieval but not after its end (but see Finn & Roediger^55^). In this line, Rc could be treated as a evaluation step by itself. It is important to highlight that the main difference between the testing effect and the reconsolidation process emerges from their research tradition. The former comes from the cognitive psychology and the latter from the neurobiology field. In this sense, Nader and Hardt^56^ proposed the reconsolidation process as the mechanism in charge of memory malleability becoming the bridge between both traditions. Another alternative interpretation could be the interference during retrieval. Based on the retrieval induced forgetting effect (RIF), Norman and coworkers developed a broad model to explain such effect^57^. Thus, they suggested that partial cued retrieval can trigger weakening of competing items. Assuming this premise, in these experiments the Rc condition could be consider a cued retrieval item which, in turns, weakens the retrieval of Rw and Nr items. In general RIF is reported for associations that belong to the same semantic category^58^, in this report pairs were not separate in semantic categories but it might be considered that all words share the same group because they are nouns and presented together in the first session. If this effect is at play during the reminders presentation, it appears clearly on Nr at both testing intervals but not on Rw, and may add to the improvement related to the strengthening by reconsolidation. Future experiments with separated treatments for each groups would reveal the presence of RIF on the Nr items in comparison with and independent group with retrieval cued items. We also consider the competition associated exclusively with the testing session during retrieval. In this sense a stronger memory would affect the expression of a weaker one^59^. This effect would reinforce the expression of the strengthened Rc items at both short and long term test, and for Rw only 5 days after training. All in all, these alternative explanations complement but not exclude our proposal based on the reconsolidation process.

Traditionally, memory enhancement has been studied behaviorally (memory precision and persistence) or by means of BOLD activation levels in specific brain regions. Here, using an after-effect whole brain network analysis, we show that reconsolidation imprint a more connected network compared with memories that were retrained or untreated. For the first time, we found a novel method to elucidate the neural footprint associated with a critical feature of memory reconsolidation.

## Materials and Methods

### Participants

Seventy-seven healthy undergraduate and graduate students from Buenos Aires University volunteered for the study (42 women). Their ages ranged from 18 to 35 years. Forty-three subjects participated in the behavioral study, and thirty-four subjects were evaluated in an fMRI scanner. All participants had to reach fifty percent of correct responses at training. The data from 24 subjects were excluded from analysis because they did not reach the learning level (nine), because they did not finish the entire experiment (ten), or because they moved during scan acquisition (five). The criterion used for movement tolerance was up to 2 mm, which corresponds to the size of half a voxel of the original acquisition dimensions.

Before their participation, all subjects signed a written informed consent form. Both the protocol and the consent were previously approved by the Ethics Committee of the Fundación para la Lucha contra las Enfermedades Neurológicas de la Infancia (FLENI) in accordance with the Declaration of Helsinki.

### Experimental Design

**Figure 5:**
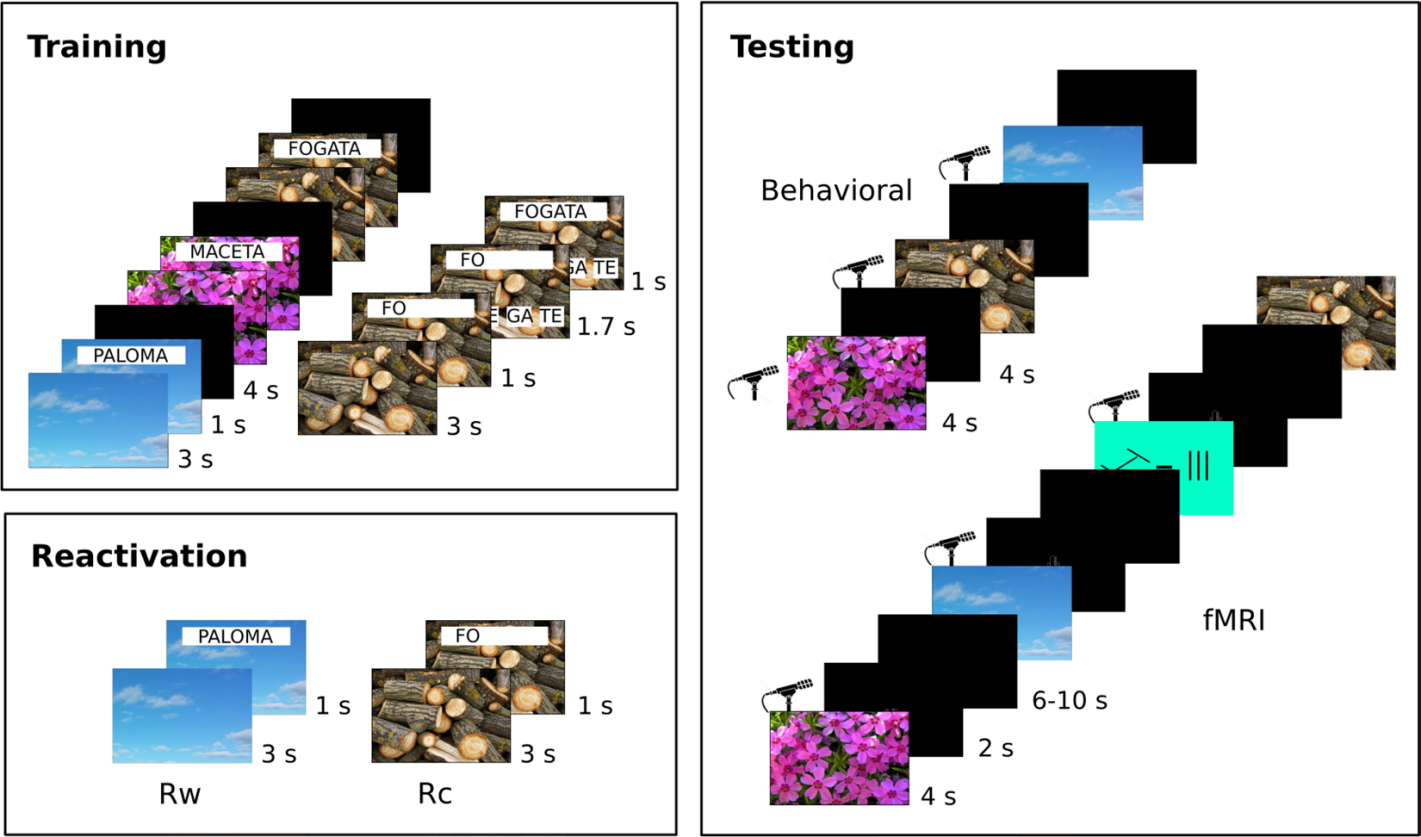
Experimental Design. Training Session. *(Upper-left panel)* The presentation of each image is followed by the presentation of the image plus the associated word. Once the 36 image-word pairs were shown each image appeared followed by the image plus the first syllable of the associated word. Then, to complete the word, 4 options were given at the bottom of the screen. Reactivation Session. *(Left-bottom panel)* There were three reminder conditions. Both the cue reminder (Rc) and the word reminder (Rw) displayed the picture, the Rc condition superimposed the picture with the first syllable (incomplete reminder) and the Rw superimposed the entire word (complete reminder). The third condition was the no reminder one (Nr), neither the picture nor the word was shown. Testing Session. *(Right panel)* In the behavioral experiment, participants had to say the associated word aloud during the presentation of each image. In the fMRI experiment, the testing session consisted of the presentation of the 36 picture followed by a microphone image symbolizing the moment to respond the word associate aloud; interspersed with the learned pictures we included control images showing two sets of lines where the participant had to answer if the two sets were different or equal. Photos of fire woods http://miro.openphoto for http://openphoto.net/ and flowers http://miro.openphoto for http://openphoto.net/ were taken from https://openphoto.net/ a free copyright’s photo bank. These images are shared under a CC-BY Share Alike license (https://creativecommons.org/) and were not modified.

We adapted the protocol performed in Forcato *et al*.^26^. The experiments consisted of three sessions, Fig. 5. The first two sessions were separated by 48 hours. The final session was assessed five or fifteen days after the first session. On day 1, subjects learned a list of picture-word associations (Training). The session on day 3 consisted of the presentation of three different types of reminders of the learned list (Reactivation). On day 5 or 15, all subjects were tested (Testing). The responses were quantified according to the type of reminders.

#### Learning task

The learning task consisted of associating 36 pictures with 36 Spanish words (i.e., a picture of sky associated with the word “PALOMA”, pigeon). The words were nouns with three syllables and six letters. Each word started with a different syllable. The picture-word pairs (items) were not directly related in a semantic dimension, and they did not share the same category. The pictures and the words were the same as those used in Forcato *et al*.^26^.

#### Type of experiments and experimental groups

We performed two separate experiments. The aim of the behavioral experiment was to compare the persistence of a memory strengthened by reconsolidation or retraining. To this aim, we carried out a behavioral experiment where the testing session was performed five days (day 5) or fifteen days (day 15) after training. The purpose of the second experiment was to explore the neural correlates imprinted in the brain by having undergone a reactivation that strengthened memory. Therefore, we performed both testing sessions (day 5 and day 15) in an fMRI scanner. Participants were randomly assigned to each experimental group.

#### Training

The training day had two parts. First, on a computer, each picture was presented for 3 seconds followed by the associated word for 1 second. After that, a black screen was shown for 3 seconds followed by the next item, until all 36 items were completed. The presentation order was randomized. Second, to evaluate the association accuracy, the presentation was repeated but with a different structure; each picture was shown for 3 seconds but only the first syllable of the word was overlaid for 1 second (Fig. 5, Training). Then, four possible syllable options appeared to complete the entire word at the bottom of the screen. Participants had 1.7 seconds to choose the two correct syllables by pressing four available keys of the keyboard with the right hand. The first key represented the first selected option on the monitor, the second key the second option, and so on. If subjects answered correctly, the word was kept in black for 1 second; if the subjects answered incorrectly, or did not respond in time, the correct answer appeared in red for 1 second. The training lasted 15 minutes. All answers were recorded.

#### Reactivation session

The 36 items were divided in three conditions or reminders (cue, word and no reminder), pseudorandomly assigned for each participant. The cue reminder condition (Rc, incomplete reminder) consisted of the presentation of a picture for 3 seconds followed by the presentation of the first syllable. Then, an interruption message appeared for 2 seconds followed by a red fixation cross for 5 seconds. Finally, a message indicating that the trial will continue for 3 seconds was shown. The structure of this reminder triggers the reconsolidation process^26,60^. In the word reminder condition (Rw, complete reminder), the first syllable was replaced by the entire word while maintaining the same presentation order (picture, word, interruption, fixation cross and continuation message). This reminder was similar to the training session and implied a retraining trial, reflected in a better performance in the evaluation session. The no reminder condition (Nr) was a passive condition, where neither the image nor the syllable/word were shown. The 24 items (Rc: cue reminder and Rw: word reminder conditions) were randomly interleaved till all were shown. It is important to emphasize that the options for completing the word never appeared during the whole reactivation session (Fig. 5, Reactivation). The reactivation lasted 5.6 minutes. Before the beginning of the reactivation session, all the subjects received the same instruction to complete the word according to the options given on the screen.

#### Testing

The 36 items were evaluated in the testing session.

**Behavioral experiment** (n = 26)

Day 5 group (n = 14): Subjects were trained (day 1), received the reactivation (48 hours later) and were then tested five days after training. The whole experiment was conducted in an experimental room at the University of Buenos Aires.

Day 15 group (n = 12): Subjects were trained (day 1) and, received the reactivation (48 hours later), but their evaluation session was assessed fifteen days after training.

In both groups, each picture was presented for 4 seconds, and at that moment, subjects had to say the associated word aloud. The inter-trial interval was 4 seconds. The answers of the participants were recorded using a microphone. The testing session lasted 4.8 minutes (Fig. 5, Testing).

**fMRI experiment** (n = 27)

Day 5 group (n = 14): Subjects were trained and reactivated in the same experimental room, and the testing session was performed in an fMRI scanner at the FLENI Institute five days after training.

Day 15 group (n = 13): Subjects were trained and reactivated in the same experimental room, but they were tested fifteen days after training in the fMRI scanner.

In both groups, each picture was presented for 4 seconds, then a black screen with a picture of a microphone was shown for 2 seconds, allowing to respond in loud voice the associated word (Fig. 5, Testing). The inter–trial interval was between 6 and 10 seconds (assigned by a Gaussian distribution, mean = 8, standard deviation = 1). We also added 12 filler pictures located between the 36 items. Each filler picture had a colored background and two groups of three lines with a tilt. The filler picture was presented for 4 seconds followed by a black screen with the picture of the microphone for 2 seconds, where participants had to say if the two groups of lines were similar or different. The session lasted 11.8 minutes. Participants were instructed to speak every time the microphone picture was shown. If they did not know the associated word, they had to say, “I cannot remember”.

### Experimental setup

The paradigm was designed and presented using homemade software in MATLAB 7.5 (Mathworks Inc., Sherborn, MA, USA) with the Psychtoolbox toolkit^61^. During the testing session in the scanner, the subjects lay inside the bore of the magnet. The subjects viewed a screen that displayed the paradigm through a mirror mounted on the head coil. The stimuli were presented by a computer outside the scanner. The subjects were instructed to answer the associated words (or to say,”I cannot remember”) in loud voice. To verify memory performance of the fMRI experiment, after the evaluation session, participants had to fill out a written form reporting the words said inside the scanner.

### Statistical Analysis for the Behavioral Data

The differences between the correct responses at testing and at training (retention) were analyzed by an ANOVA for repeated-measures with “reminder condition” (Rc, Rw, Nr) as within-subjects factor and “time” (Day 5 and Day 15) as the between-subjects factor. When sphericity was not accomplished, Greenhouse−Geisser correction was applied. Then, the ANOVA was followed by post-hoc pairwise comparison using the Bonferroni correction for the main effects.

### fMRI data and image processing

#### Image acquisition

A 3-Tesla General Electric Signa HDxt (GE Medical Systems, Milwaukee, Wisconsin, USA) scanner was used to acquire all the images. An 8-channel head coil was used to receive signal intensity. A three-plane localizer image was initially obtained to facilitate the positioning of the transverse sections parallel to the anterior-posterior commissure line. For fMRI, an interleaved ascendent T2*-weighted gradient echo EPI sequence was used to cover the whole brain (TR/TE = 2500/30 ms; acquisition matrix size = 64 × 64; FOV = 24 cm; slice thickness = 4 mm, with zero spacing between images; in-plane resolution = 3.75 × 3.75 mm2; 30 contiguous sections). The total acquisition time was 12 minutes, including 5 dummy scans to allow for T1 saturation effects that were discarded from the analysis. A total of 408 volumes were acquired. For anatomic reference, a high resolution T1-weighted 3D fast SPGR-IR was used (TR = 6.604 ms, /TE = 2.796 ms, /TI = 450; parallel imaging (ASSET) acceleration factor = 2; acquisition matrix size = 256 × 256; FOV = 24 cm; slice thickness = 1.2 mm; 120 contiguous sections).

#### Image Processing

Data were analyzed using Statistical Parametric Mapping (SPM8, Wellcome Department of Cognitive Neurology, University College, London, UK) implemented in MATLAB (Mathworks Inc., Sherborn, MA, USA). A interleaved slice-timing correction was applied to each volume. The imaging time series was realigned to the first image to correct for the subjects motion during acquisition and spatially normalized using a Montreal Neurological Institute reference brain^62^. The spatially normalized volumes consisted of 2 mm^3^ voxels. These data were subsequently smoothed with an isotropic Gaussian kernel of 8 mm at full width half-maximum^63^.

We modeled the individual BOLD signal with the canonical hemodynamic response function using the general linear model (GLM) with nine regressors of interest: the pictures presented in the three conditions, the filler pictures, the microphone screens in the three conditions, the microphone of the filler picture and the black screen of intervals. The design matrix also included 6 parameters for head movement corrections as regressors of no interest. We only analyzed the regressors of the pictures of the three conditions (Rc, Rw and Nr). The pauses between trials were used as baseline in the ROI Analysis.

### ROI Analysis

We quantified the activity based on regions of interest (ROIs, derived from AAL Atlas^64^). We considered the β-values for each region as the difference between the mean signal of the voxels for each condition and the baseline activity (pause between trials). We considered 32 ROIs associated with memory retrieval processes and testing effect reports^22–24,26,35,36^ and/or anatomical regions that showed significant activity in more than ten voxels using t-maps of the whole brain analysis Rw > Rc, Rc > Nr, Rw > Nr and Rw < Rc, Rc < Nr, Rw < Nr, Supplementary Fig. S1 and Table S2. To simplify the results, we grouped the individual regions into seven big regions (1. hippocampus; 2. parahippocampus; 3. prefrontal cortex: frontal inferior triangularis, frontal superior medial, frontal superior, frontal middle and frontal middle orbital; 4. parietal lobe: supramarginal, angular and parietal superior; 5. temporal lobe: temporal superior, temporal middle and temporal inferior; 6. posterior cingulum; 7. anterior cingulum). To perform the analysis, we used MarsBaR (MARSeille Boîte À Région d’Intérêt), a toolbox for SPM that provides routines for region of interest analysis. Features include region of interest definition, combination of regions of interest with simple algebra, extraction of data for regions with and without SPM preprocessing (scaling, filtering), and statistical analyses of ROI data using the SPM statistics machinery^65^.

We performed one-way ANOVA for the seven regions, with reminder condition as the within-subjects factor (with three levels: Rc, Rw and Nr), using a threshold of α = 0.05. Then, we performed Bonferroni’s multiple comparisons correction (α = 0.05).

### Connectivity analysis

We generated individual connectivity matrices to study the temporal correlation between areas during the conditions. To assess the functional connectivity (FC) of a short-events design, we used the beta correlation methods as described in Mäki-Marttunen *et al*.^66,67^. This method assumes that the extent to which two brain voxels interact during a given task condition is reflected in the extent to which their respective beta series from that condition are correlated. A GLM was modeled to each picture (4 seconds) as a separate event and concatenated for each condition (Rc, Rw and Nr). In this way, we obtained a time series per voxel, per condition and participant. Each time series was filtered with a bandpass between 0.01 and 0.25 Hz using the REST software (a toolkit for resting-state fMRI^68^). To construct the networks matrices, we parcellated the whole brain into 90 discrete anatomical regions in accordance with the automated anatomical labeling (AAL)^64^ and performed Pearson’s correlation analysis between the mean beta series of each pair of regions, resulting in a 90 × 90 matrix per subject per condition. We obtained an interregional association matrix $C={c}_{i,j}\,$with $i,j=1,2,3,\mathrm{.}.,90$. Then, we applied an arc-hyperbolic tangent transform^67^ to the correlation coefficients of all brain regions ${z}_{i,j}=\text{arctanh}({c}_{i,j})$.

Finally, we averaged the 90 × 90 cross correlation matrices per condition and calculated the grand mean matrix ${r}_{i,j}=\sum _{1}^{N}{z}_{i,j}\,$with N being the number of participants. To explore the topological properties of these networks, we analyzed the mathematical features for different threshold values assuming that two regions (i and j) were connected (linked) if *r*_*i,j*_ was equal to or greater than this threshold. We defined 6000 threshold values between the minor coefficient *r*_*i,j*_ and 0.6. To complete the definition of a network, we named nodes to the sites with nonzero number of links. In other words, we obtained an undirected and unweighted graph per condition for each threshold value. To characterize the properties of the networks, we choose to analyze two Graph Theory measures for each threshold value.Graph Theory measures

Average degree (<k>): The network average degree is commonly used as a measure of graph density^29,30^. It is the number of links connecting a regional node to the rest of the brain graph. If *k*_*i*_ is the number of links for the node i (degree), the average degree is defined by $\langle {k}_{i}\rangle =\frac{1}{n}\sum _{j}^{n}{k}_{j}$with n being the number of nodes.

Clustering coefficient (C): The clustering coefficient captures the degree to which the neighbors of a given node link to each other. The clustering coefficient measures the local link density by counting the number of complete triangles per node^29^. It reflects the prevalence of clustered connectivity^32^. The local clustering coefficient is between 0 and 1; it is 0 when none of the neighbors link to each other and 1 when all the nodes link to each other. The global clustering coefficient, or the clustering coefficient is defined as the average of the local measures.

To analyze if the topological features of the networks were due to real effects of the experiment, we calculated C for Erdos-Renyi random graphs with the same number of nodes and links with probability of attachment 0.5 per condition per threshold. We performed a statistical test to compare the C for the three experimental conditions and C for the random graphs per threshold value. *p* values are obtained by calculating the distribution of C for 1000 random graphs per threshold and then comparing these with C of each condition (critical alpha-level was 0.01).

## Supplementary information

Whole brain t- maps
