# Author Correction: Retrieval of retrained and reconsolidated memories are associated with a distinct neural network

**DOI:** 10.1038/s41598-019-42488-0

**Published:** 2019-04-10

**Authors:** Luz Bavassi, Cecilia Forcato, Rodrigo S. Fernández, Gabriela De Pino, María E. Pedreira, Mirta F. Villarreal

**Affiliations:** 1 Universidad de Buenos Aires, Facultad de Ciencias Exactas y Naturales, Departamento de Física, Ciudad de Buenos Aires, Argentina; 2 CONICET-Universidad de Buenos Aires, Instituto de Fisiología, Biología Molecular y Neurociencias (IFIBYNE), Ciudad de Buenos Aires, Argentina; 3 Unidad Ejecutora de Estudios de Neurociencias y Sistemas Complejos, CONICET, Universidad Nacional Arturo Jauretche Hospital de Alta Complejidad en Red El Cruce “Néstor Kirchner”, Av. Calchaqui 6200, (1888), Florencio Varela, Argentina; 4 Universidad de Buenos Aires, Facultad de Ciencias Exactas y Naturales, Ciudad de Buenos Aires, Argentina; 5 Laboratorio de Neuroimágenes, Departamento de Imágenes, FLENI, Montañeses 2325, Ciudad de Buenos Aires, C1428AQK Argentina; 6 Centro Universitario de Imágenes Médicas (CEUNIM), Escuela de Ciencia y Tecnología, Universidad Nacional de San Martín, Buenos Aires, Argentina; 7 INAAC, FLENI, Montañeses 2325, C1428AQK Ciudad de Buenos Aires, Argentina; 8 CONICET, Ciudad de Buenos Aires, Argentina; 9 Present Address: Instituto de Fisiología, Biología Molecular y Neurociencias, Facultad de Ciencias Exactas y Naturales, Universidad de Buenos Aires, Ciudad Universitaria (C1428EHA), Ciudad de Buenos Aires, Argentina

Correction to: *Scientific Reports* 10.1038/s41598-018-37089-2, published online 28 January 2019

The Author Contributions section in this Article is incomplete.

“L.B., C.F., R.F., G.D.P., M.E.P. and M.V. contributed to the conception and design of the experiment. L.B., R.F., C.F., M.E.P. and M.V. wrote the main manuscript text. L.B., G.D.P. and M.V. contributed to the analysis of data. L.B. prepared figures. L.B., G.D.P., R.F. and M.V. collected data. All authors reviewed the manuscript”.”

should read:

“L.B., C.F., R.F., G.D.P., M.E.P. and M.V. contributed to the conception and design of the experiment. L.B., R.F., C.F., M.E.P. and M.V. wrote the main manuscript text. L.B., G.D.P. and M.V. contributed to the analysis of data. L.B. prepared figures. L.B., G.D.P., R.F. and M.V. collected data. All authors reviewed the manuscript. L.B. and C.F. contributed equally as first authors; R.F. and G.D.P contributed equally as second authors; M.E.P. and M.V. contributed equally as last authors.”

