# Supplementary material for: Retrieval of retrained and reconsolidated memories are associated with a distinct neural network
Source: Sci Rep. 2019 Jan 28;9:784. doi: 10.1038/s41598-018-37089-2 (PMC6349866; doi:10.1038/s41598-018-37089-2)
Supplement: Supplementary file 1 — Whole brain t- maps [file 41598_2018_37089_MOESM1_ESM.pdf]

Retrieval of retrained and reconsolidated memories are associated with a distinct neural network

Luz Bavassi[a,b,1,#,*], Cecilia Forcato[c,1], Rodrigo S. Fernández[d,b,2], Gabriela De Pino[e,f,g,2], María E. Pedreira[d,b,3], Mirta F. Villarreal[a,g,h,3]

[a] Universidad de Buenos Aires, Facultad de Ciencias Exactas y Naturales Departamento de Física, Ciudad de Buenos Aires,Argentina.

[b] CONICET-Universidad de Buenos Aires, Instituto de Fisiología, Biología Molecular y Neurociencias (IFIBYNE), Ciudad de Buenos Aires, Argentina.

[c] Unidad Ejecutora de Estudios de Neurociencias y Sistemas Complejos, CONICET, Universidad Nacional Arturo Jauretche Hospital de Alta Complejidad en Red El Cruce "Néstor Kirchner",Av. Calchaqui 6200 , (1888) Florencio Varela, Argentina.

[d] Universidad de Buenos Aires, Facultad de Ciencias Exactas y Naturales, Ciudad de Buenos Aires, Argentina.

[e] Laboratorio de Neuroimágenes, Departamento de Imágenes, FLENI, Montañeses 2325, Ciudad de Buenos Aires (C1428AQK), Argentina.

[f] Centro Universitario de Imágenes Médicas (CEUNIM), Escuela de Ciencia y Tecnología, Universidad Nacional de San Martín, Buenos Aires, Argentina.

[g] INAAC, FLENI, Montañeses 2325, Ciudad de Buenos Aires (C1428AQK),Argentina.

[h] CONICET, Ciudad de Buenos Aires, Argentina.

[1] Contributed equally to this work; [2] Contributed equally to this work; [3] Contributed equally to this work

#Corresponding author & *Lead contact: Luz Bavassi. Instituto de Fisiología, Biología Molecular y Neurociencias, Facultad de Ciencias Exactas y Naturales, Universidad de Buenos Aires, Ciudad Universitaria, (C1428EHA), Buenos Aires, Argentina.

Mail: luzbavassi@gmail.com

PH:+541134344738.

Supplementary information

We performed individual contrasts between different conditions (Rw > Rc; Rc > Nr; Rw> Nr and Rw<Rc; Rc<Nr; Rw<Nr) for each subject. Then, those contrasts were taken into a second-level analysis (one sample t-test) to find the group response. The results are shown at a threshold of $p<0.001$ uncorrected with a cluster size of $k=10$. We analyzed the t-maps between the three conditions for both evaluation sessions to explore the neural imprint of a memory that was strengthened Supplementary Figure S1 and Supplementary Table S2. On day 5, the Rw condition had higher activity than the Rc condition (Rw>Rc) and the Nr condition (Rw>Nr). Most of the significant clusters ($p<0.001$ and $k=10$) were located in the left hemisphere. Particularly, the differences appeared in parietal regions when we contrasted Rw (retrained condition) with Rc (the reconsolidation treatment) or Rw and Nr conditions. In this last comparison, we also found differences in frontal regions, temporal lobe, hippocampus and posterior cingulum, Table 1. Moreover, we also found a significant cluster centered on the right posterior cingulum for Rw>Rc ($p<0.001$, $t= 4.61$). We did not find any significant clusters in the opposite comparisons (Rw<Rc or Rw<Nr) or between Nr and Rc. At first glance, the t-maps obtained in the testing session on day 15 (Supplementary Figure S1 and Supplementary Table S2) showed fewer significant clusters than day 5 ($p<0.001$ and $k=10$). There were differences when we compared Rc>Nr, with a significant cluster in the left parietal lobe in the postcentral gyrus ($p<0.001$, $t= 8.43$), Supplementary Table S2. We also found a significant cluster in the left temporal lobe when Rw>Nr, but we did not find differences in the left hemisphere in the Rw>Rc contrast. The results did not reveal a difference in Rc>Rw, Nr>Rw or Nr>Rc comparisons.

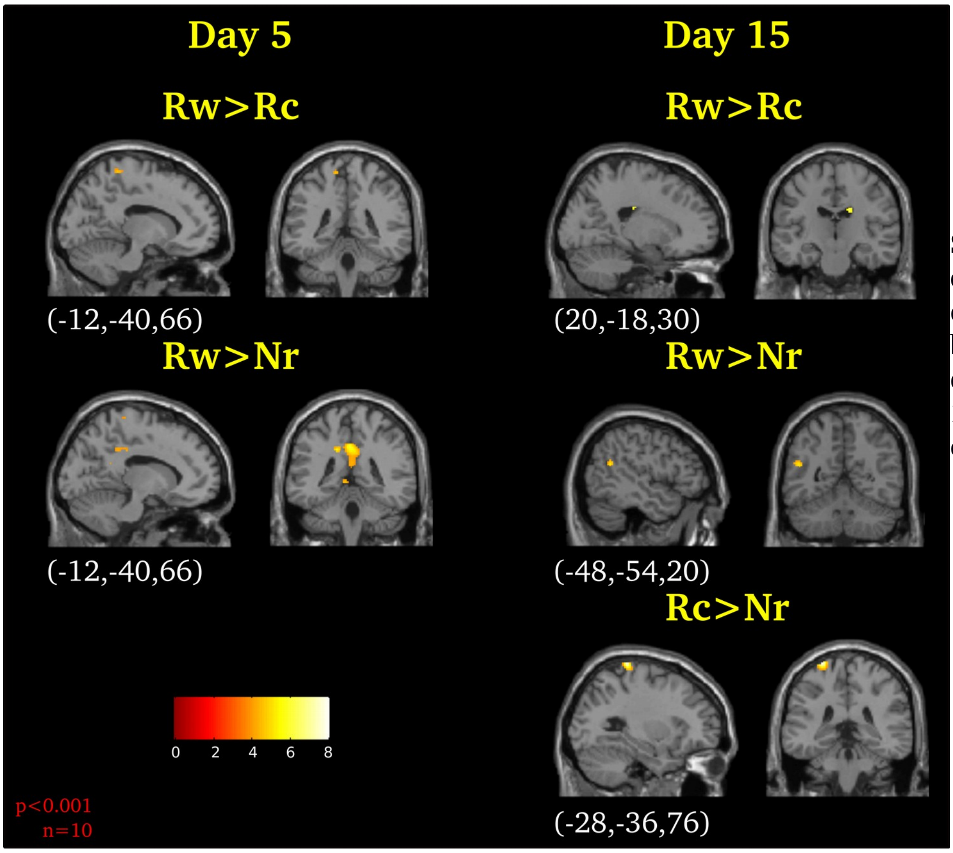

Supplementary Figure S1. fMRI experiment - t-maps on day 5 and day 15. Whole brain comparison between reminder conditions for the evaluation session on day 5 and day 15 (p<0.001 uncorrected p-value, cluster size k=10).

Supplementary TABLE S2. Significant clusters of the t-maps on Day 5 and Day 15.

Brain Area	Cluster size (n° voxels)	Cluster p	Voxel T-value	MNI Coordinates (x,y,z)		
**Day 5**						
**Rw > Rc**						
**Left Precuneus**	59	< 0.001	4.47	-12	-40	66
**Left Postcentral**			4.38	-22	-30	68
**Corpus Callosum**	15	0.006	4.28	-18	-50	18
**Rw > Nr**						
**Left Middle Cingulum**	390	< 0.001	5.80	-2	-40	38
**Left Posterior Cingulum**			5.80	-16	-42	36
**Right Posterior Cingulum**			4.61	-4	-40	18
**Left Posterior Cingulum**	111	< 0.001	5.61	-11	-49	23
**Left Hippocampus**	11	0.017	5.56	-16	-12	-18
**Left Paracentral**	67	< 0.001	5.08	-2	-30	68
**Left Postcentral**	86	< 0.001	4.88	-22	-30	72
**Left Thalamus**	21	0.002	4.52	-18	-26	16
**Left Lingual**	11	0.017	4.46	-8	-40	0
**Left Angular**	13	0.010	4.44	-38	-48	28
**Left Temporal Inferior**	11	0.017	4.34	-48	-42	-22
**Day 15**						
**Rw> Rc**						
**Right Caudate**	25	< 0.001	6.08	18	-19	26
**Rw>Nr**						
**Left Middle Temporal**	29	< 0.001	5.14	-48	-54	20
**Rc>Nr**						
**Left Postcentral**	87	< 0.001	8.43	-48	-36	7
